# Classification tree for detection of single-nucleotide polymorphism (SNP)-by-SNP interactions related to heart disease: Framingham Heart Study

**DOI:** 10.1186/1753-6561-3-s7-s83

**Published:** 2009-12-15

**Authors:** Li Yao, Wenjun Zhong, Zhumin Zhang, Matthew J Maenner, Corinne D Engelman

**Affiliations:** 1 Department of Human Development and Family Studies, University of Wisconsin-Madison, 1300 Linden Drive, Madison, Wisconsin 53706, USA; 2 Department of Population Health Sciences, University of Wisconsin-Madison, 610 Walnut Street, Madison, Wisconsin 53726, USA; 3 Department of Nutritional Sciences, University of Wisconsin-Madison, 1415 Linden Drive, Madison, Wisconsin 53706, USA

## Abstract

The aim of this study was to detect the effect of interactions between single-nucleotide polymorphisms (SNPs) on incidence of heart diseases. For this purpose, 2912 subjects with 350,160 SNPs from the Framingham Heart Study (FHS) were analyzed. PLINK was used to control quality and to select the 10,000 most significant SNPs. A classification tree algorithm, *Generalized, Unbiased, Interaction Detection and Estimation *(GUIDE), was employed to build a classification tree to detect SNP-by-SNP interactions for the selected 10 k SNPs. The classes generated by GUIDE were reexamined by a generalized estimating equations (GEE) model with the empirical variance after accounting for potential familial correlation. Overall, 17 classes were generated based on the splitting criteria in GUIDE. The prevalence of coronary heart disease (CHD) in class 16 (determined by SNPs rs1894035, rs7955732, rs2212596, and rs1417507) was the lowest (0.23%). Compared to class 16, all other classes except for class 288 (prevalence of 1.2%) had a significantly greater risk when analyzed using GEE model. This suggests the interactions of SNPs on these node paths are significant.

## Introduction

Coronary heart disease (CHD) is a common and complex disease that is likely to involve many different genes interacting with each other and with the environment. Many studies published so far have only considered single-nucleotide polymorphisms (SNPs) in a single gene, with little consideration given to the interactions between genes. The Genetic Analysis Workshop 16 (GAW16) Framingham Heart Study (FHS) dataset provided through the database of Genotype and Phenotype (dbGaP) includes data for 550,000 SNPs and provides us with a unique opportunity to investigate this issue [1].

Genome-wide association studies (GWAS) systematically investigate SNPs in the entire human genome. This process allows identification of SNPs that may be associated with the disease of interest. Although GWAS are often criticized for not being hypothesis driven and can be described as data mining, they have identified unexpected and unpredictable genetic links that have advanced scientific knowledge substantially on heart disease as well as other diseases [2-6].

Challenges of statistical analysis of GWAS data have been addressed [7]. The most difficult problems associated with GWAS analysis are: 1) how to handle extremely large data sets, often times with >10 gigabytes and 2) how to deal with a large *p*, small *n *problem due to the immense number of SNPs accompanied by a relatively small sample size. Different methods have been proposed to reduce the dimension of the data. One method is to use machine-learning approaches to select SNPs that could best explain a phenotype. A classification-tree algorithm called GUIDE [8], which stands for *Generalized, Unbiased, Interaction Detection and Estimation*, was employed in the present study. It is specifically designed to eliminate variable selection bias, a problem that can undermine the reliability of inferences from a tree structure. The algorithm of GUIDE is unbiased and is sensitive to local interactions during split selection. The FHS data from the GAW16 (Problem 2) was utilized to examine genes associated with CHD.

## Methods

### Data set and initial data quality checking

The FHS is a family-based study that enrolled three generations. CHD is defined as any of the following: recognized myocardial infarction diagnosed through an EKG or enzymes, coronary insufficiency, or death attributed to CHD. The third generation was excluded from the present analysis because most of them were too young to develop CHD, creating the potential for misclassification of the outcome. For the remaining 2941 study subjects, if he/she was ever diagnosed having CHD during the entire study period, this subject was classified as having CHD (case). Otherwise, the subject was classified as CHD free (control).

Dense genotyping for each study subject was performed using approximately 550,000 SNPs across 22 autosomal chromosomes (GeneChip Human Mapping 500 k Array Set and the 50 k Human Gene Focused Panel). Affymetrix conducted all genotyping for the FHS, using the 250 k Sty, 250 k Nsp, and the supplemental 50 k platforms. Quality control checks for the SNPs were performed in PLINK software [9] (PLINK v1.03, http://pngu.mgh.harvard.edu/purcell/plink). SNPs with >5% missing genotypes (*n *= 31,975) and with minor allele frequency <5% (*n *= 111,290) were excluded. Another 20,646 SNPs failed Hardy-Weinberg equilibrium test (*p*-value < 0.001). Subjects with >5% of SNPs missing (*n *= 29) were excluded. The remaining 2912 subjects (228 cases and 2684 controls) with 350,160 SNPs were included in subsequent analyses.

### GUIDE

The tree algorithm GUIDE, version 7.0, was used for building a classification tree [8]. GUIDE develops a tree by three steps: 1) a chi-square test selects the most significant split variable to split a node; 2) the split set is selected to minimize a node impurity measure (the impurity measure in GUIDE includes entropy and Gini index); 3) Steps 1 and 2 are recursively repeated until too few observations are in each node. After building a complete tree, three methods including cross-validation pruning (default), test-sample pruning, and no pruning are used to decide how much of the tree to retain. The criteria for pruning is to minimize unbiased estimate of misclassification cost. GUIDE allows fast computational speed, natural extension to data sets with categorical variables, and direct detection of local two-variable interactions. It has four useful properties: i) negligible selection bias; ii) sensitivity to curvature and local pairwise interactions between regressor variables; iii) inclusion of categorical predictor variables; and iv) choice of three roles for each ordered predictor variable: split selection only, regression modeling only, or both.

GUIDE can process a large number of SNPs in one run. However, it is still not feasible to run the entire data set with 10 GB and 350,160 SNPs at one time due to computation limitations (i.e., GUIDE stopped running, potentially due to a read buffer that is too small). To overcome this problem, the top 10,000 SNPs associated with CHD using a chi-square test implemented in PLINK were analyzed in GUIDE.

### Evaluation of classes identified by GUIDE using GEE model

Current tree algorithms cannot handle dependent data such as that in the FHS, where family members are dependent. To accommodate this limitation, study subjects were treated as independent using GUIDE. The classes of SNPs identified by GUIDE were re-evaluated using a generalized estimating equations (GEE) model with the empirical variance to account for potential familial correlation.

## Results

### Descriptive statistics

Descriptive statistics for the individuals in Generations 1 and 2, as well as those for the combined data set are shown in Table 1.

**Table 1:** Descriptive statistics of selected traits by generation: range, percentage distribution or mean, and standard deviation at baseline

Trait^a^	Generation 1 (*n *= 356)			Generation 2 (*n *= 2556)			Generation comparison	Overall (*n *= 2912)		
				
	Range	Mean	SD	Range	Mean/%	SD		Range	Mean/%	SD
Age	29-54	34.87	3.79	5-59	33.72	9.26	t = 2.31^b^	5-59	33.86	8.78
Body Mass Index	16.7-36.0	23.81	3.27	13.5-51.1	24.94	4.10	t = -4.95^c^	13.5-51.1	24.80	4.03
SBP	90-160	123.01	13.45	78-200	119.13	14.24	t = 4.86^c^	78-200	119.60	14.20
DBP	50-105	77.85	9.35	48-120	77.16	9.97	t = 1.23	48-120	77.25	9.90
Cholesterol	129-339	191.91	37.28	101-388	190.13	36.23	t = 0.68	101-388	190.26	36.30
Cigarettes	0-50	7.58	10.71	0-88	7.60	12.09	t = -0.03	0-88	7.60	12.00
Smoke	0-2			0-2			χ^2 ^= 25.97^c^	0-2		
	0	44.09%		0	41.27%			0	41.46%	
	1	5.38%		1	20.01%			1	19.01%	
	2	50.54%		2	38.72%			2	39.52%	
Diabetes	0-1			0-1			χ^2 ^= 2.24	0-1		
	0	92.13%		0	89.59%			0	89.90%	
	1	7.87%		1	10.41%			1	10.10%	

^a^SBP, systolic blood pressure (mm Hg); DBP, diastolic blood pressure (mm Hg); Cholesterol, fasting × total cholesterol (mg/dl); Cigarettes, number of cigarettes smoked per day; Smoke, smoking status (0, never; 1, former; 2, current); Diabetes, diabetes status (0, No; 1, Yes).

^b^*p *< 0.05

^c^*p *< 0.001

### Classification tree build by GUIDE

**Figure 1:**
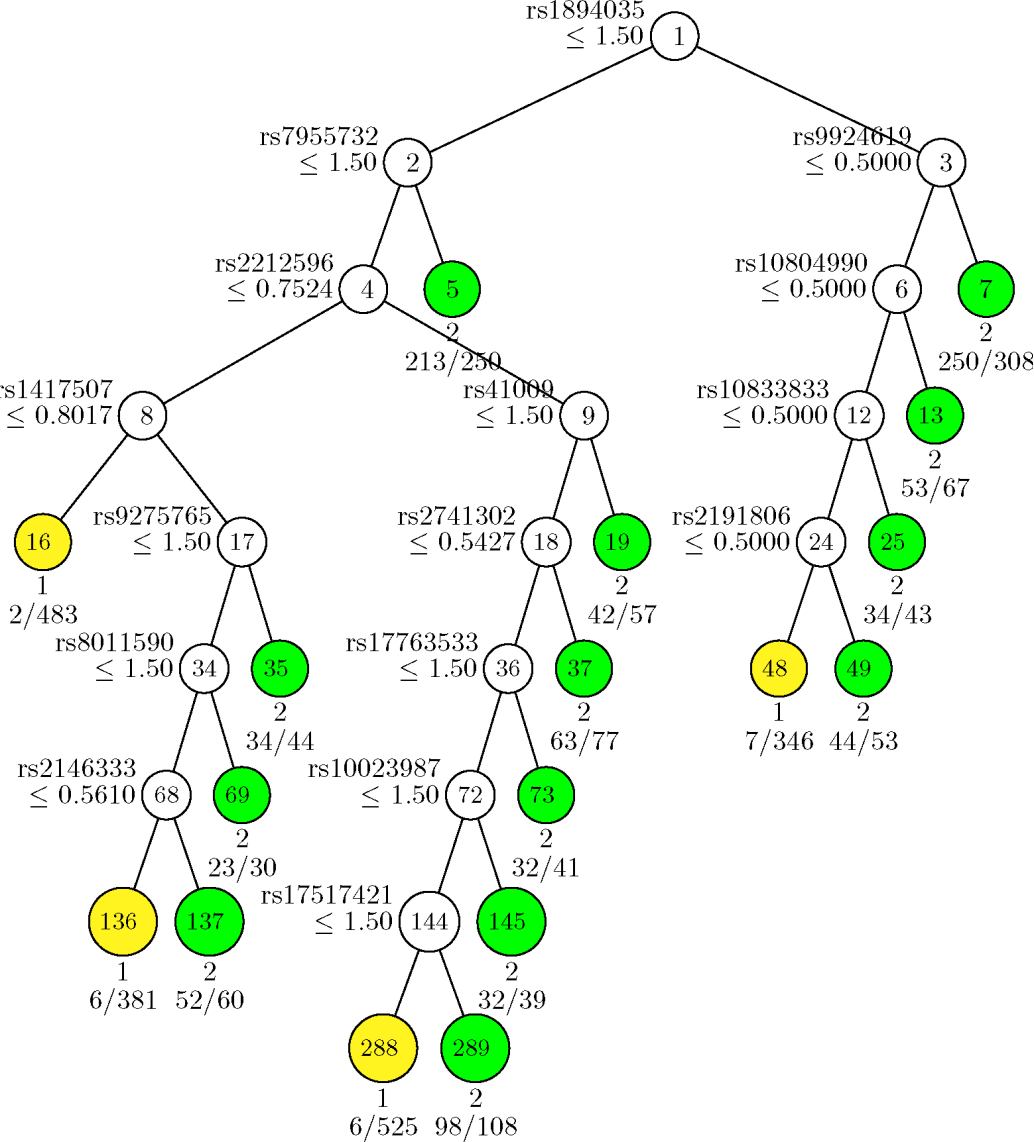
**Classification tree generated by GUIDE**. For each SNP, an additive genetic model with respect to the minor allele was used. At each intermediate node, a case went to the left child node if and only if the condition was satisfied. The classification tree generated by GUIDE had 17 terminal nodes (yellow or green). Node numbers were labeled at the terminal nodes. Nodes in yellow were controls (coded as 1) and nodes in green were cases (coded as 2). There were 228 cases in the sample. Predicted class and number of errors divided by number of cases are given beneath each terminal node. The current tree in Figure 1 pruned by ten-fold cross-validation has the smallest misclassification cost.

10,000 candidate SNPs pre-selected by PLINK were analyzed in GUIDE. The final classification tree had a total of 33 nodes, 17 of which were terminal nodes (Figure 1). The 16 SNPs that determined the splits are described in Table 2.

**Table 2:** Functions of 16 SNPs that determined the splits in the classification tree

SNP	Alleles	MAF^b^	Chromosome	Position	Gene	Function	*p*-value^c^
rs2741302	A^a^/C	0.179582	2	232,936,077			0.0245
rs41009	A^a^/G	0.134071	2	8,012,609			0.0009
rs10023987	C^a^/T	0.196187	4	98,109,876			0.0068
rs10804990	A/G^a^	0.381304	4	6,970,562	TBC1D14 TBC1 domain family member 14	intron	0.0253
rs1417507	T^a^/C	0.276753	6	50,324,791			0.0146
rs2146333	A^a^/C	0.188192	6	36,564,996	KCTD20 potassium channel tetramerization domain containing 20	3'UTR	0.01703
rs9275765	A^a^/T	0.215867	6	32,797,302			
rs2191806	C^a^/G	0.182042	7	78,581,862	MAGI2 membrane associated guanylate kinase, WW and PDZ domain containing 2	intron	0.0169
rs17517421	A^a^/G	0.113776	8	18,410,095			0.0007
rs10833833	A^a^/G	0.123616	11	3,355,238	ZNF195 zinc finger protein 195	intron	0.0092
rs1894035	C^a^/T	0.311808	12	50,932,021	KRT86 keratin 86	intron	0.02598
rs7955732	G^a^/T	0.403444	12	71,011,262	TRHDE thyrotropin-releasing hormone degrading enzyme	intron	0.02056
rs8011590	C^a^/G	0.083025	14	106,109,086			0.01665
rs9924619	C^a^/G	0.115006	16	28,924,029			0.00072
rs17763533	C^a^/T	0.214637	17	41,273,970			0.01929
rs2212596	A^a^/C	0.49754	21	38,890,404	ERG v-ets erythroblastosis virus E26 oncogene homolog (avian)	intron	0.01421

^a^Minor allele

^b^MAF, minor allele frequency

^c^*p*-values from single-marker analysis by PLINK

### Risk of CHD in each of 17 classes of classification tree

Patients with missing values at one or more of the 16 SNPs were excluded (*n *= 160). The remaining 2752 patients were assigned into one of the 17 tree classes based on the splitting criteria. The prevalence of CHD for each class is presented in Table 3. The results from GUIDE were then tested in a GEE model that accounted for familial correlation. The overall test for a variable with 17 classes was significant (*p*-value < 0.0001). The prevalence of CHD in Class 16 was the lowest (0.23%, Table 3). Compared to Class 16, all the other classes except for Class 288 (prevalence of 1.2%) had a significantly greater risk of CHD, especially for Class 7, 13, 19, 73, 35, and 69, with ≥ 20% of the subjects having CHD (Table 3).

**Table 3:** Prevalence of CHD in each of 17 classes identified by GUIDE^a^

**Class No**. (Terminal No.)	No. subjects	No. CHD (%)	*p*-value^b^
16	433	1 (0.23%)	Reference
288	489	6 (1.2%)	0.12
136	367	6 (1.6%)	<0.0001
48	335	7 (2.1%)	0.04
289	106	10 (9%)	0.0004
137	56	8 (14%)	<0.0001
5	235	36 (15%)	<0.0001
49	50	8 (16%)	<0.0001
37	73	13 (18%)	<0.0001
25	42	8 (19%)	<0.0001
145	37	7 (19%)	<0.0001
7	297	58 (20%)	<0.0001
35	43	9 (21%)	<0.0001
13	65	14 (22%)	<0.0001
69	30	7 (23%)	<0.0001
73	38	9 (24%)	<0.0001
19	56	15 (27%)	<0.0001
Overall	2752	222 (8%)	---

^a^Note: GEE model deleted 160 subjects with missing SNPs, while GUIDE kept all the missing values and treated them as zero.

^b^*p*-value from the GEE model for each class compared to the reference class (16).

## Conclusion

Prevalence of CHD in people older than 45 years was estimated to be 1.6%-16.8%, depending on age and sex according to NHANES 1999-2002 data [10]. In the FHS, about 8% of the first and second generation participants had CHD. The prevalence of CHD in Class 16 (determined by SNPs rs1894035, rs7955732, rs2212596, and rs1417507) was the lowest (0.23%), while the prevalence of CHD in Class 19 (determined by SNPs rs1894035, rs7955732, rs2212596, and rs41009) was the highest (27%). The results suggest that individuals with certain combinations of genotypes are more resistant to developing CHD, while others are more susceptible. The SNPs involved in this risk gradient have not been reported in the CHD literature; they are novel candidates for future CHD research.

One important limitation of this study is that, like other machine-learning approaches, the computational capacity of GUIDE is limited by its inability to handle dependent data found in family-based studies, although the findings from GUIDE were replicated in a GEE model that accounts for familial correlation. Nevertheless, our analysis demonstrated that the unbiased selection tree algorithm, GUIDE, could be useful in reducing the number of SNPs in GWAS. It can be used to study gene-gene interactions associated with complex diseases, such as CHD. In addition, GUIDE can use any categorical or quantitative variables to do the classification (environmental or genetic).

## List of abbreviations used

CHD: Coronary heart disease; FHS: Framingham Heart Study; GAW16: Genetic Analysis Workshop 16; GWAS: Genome-wide association studies; GEE: Generalized estimating equations; GUIDE: Generalized, Unbiased, Interaction Detection and Estimation; SNPs: Single-nucleotide polymorphisms.

## Competing interests

The authors declare that they have no competing interests.

## Authors' contributions

LY participated in the design of the study, performed the main statistical analysis, and drafted the manuscript. WZ participated in the design of the study, data cleaning, and interpretation of the results, and helped to draft and revise the manuscript. ZZ participated in the design of the study, data analysis, and interpretation of the results, and drafted and revised the manuscript. MJM performed data analysis and helped revise the manuscript. CDE obtained IRB approval for the study, gained access to the data set, participated in the design of the study, and helped revised the manuscript. All authors read and approved the final manuscript.
